# Targeting the ST3 beta‐galactoside alpha‐2,3‐sialyltransferase 1 (ST3Gal1) as a potential therapeutic strategy to overcome anti‐VEGF resistance in endometrial cancer

**DOI:** 10.1002/ijgo.70292

**Published:** 2025-06-11

**Authors:** Chia‐Hao Liu, Szu‐Ting Yang, Wei‐Ting Chao, Chen‐Hao Lin, Yu‐Chieh Lee, Chiung‐Ru Lai, Shie‐Liang Hsieh, Liang‐Wei Wang, Peng‐Hui Wang

**Affiliations:** ^1^ Department of Obstetrics and Gynecology Taipei Veterans General Hospital Taipei Taiwan; ^2^ Department of Obstetrics and Gynecology National Yang Ming Chiao Tung University Taipei Taiwan; ^3^ Institute of Clinical Medicine National Yang Ming Chiao Tung University Taipei Taiwan; ^4^ Graduate Institute of Medical Sciences, College of Medicine Taipei Medical University Taipei Taiwan; ^5^ Department of Pathology and Laboratory Medicine Taipei Veterans General Hospital Taipei Taiwan; ^6^ Genomic Research Center Academia Sinica Taipei Taiwan; ^7^ Immunology Research Center National Health Research Institutes Miaoli County Taiwan; ^8^ Department of Medical Research Taipei Veterans General Hospital Taipei Taiwan; ^9^ Department of Urban Industrial Management and Marketing University of Taipei Taipei Taiwan; ^10^ Female Cancer Foundation Taipei Taiwan

**Keywords:** angiogenesis, endometrial cancer, epithelial–mesenchymal transition, ST3Gal1, VEGF‐A

## Abstract

**Objective:**

To investigate the role of ST3 beta‐galactoside alpha‐2,3‐sialyltransferase 1 (ST3Gal1) in endometrial cancer (EC) progression and its potential as a therapeutic target to enhance the efficacy of antiangiogenic treatment.

**Methods:**

ST3Gal1 expression and its clinical relevance were analyzed in EC tissues. Functional assays evaluated its effects on vascular endothelial growth factor‐A (VEGF‐A) expression, epithelial–mesenchymal transition (EMT), and cell invasiveness. Mechanistic studies, including Duolink proximity ligation assays and co‐immunoprecipitation, examined ST3Gal1–VEGF‐A interactions. ST3Gal1 was inhibited genetically or pharmacologically using soyasaponin I (SsaI), both in vitro and in xenograft models, alone or combined with bevacizumab. Angiogenic and EMT marker expression and focal adhesion kinase (FAK)/paxillin pathway activation were assessed.

**Results:**

ST3Gal1 was amplified and overexpressed in EC and correlated with advanced stage, deep myometrial invasion, and poor prognosis. It directly glycosylated VEGF‐A and activated FAK/paxillin signaling, promoting VEGF‐A expression and EMT. ST3Gal1 inhibition via SsaI reduced VEGF‐A signaling, reversed EMT marker expression, and suppressed cell migration and invasion, particularly in RL95‐2 cells. In vivo, SsaI significantly inhibited tumor growth and angiogenesis, with the most pronounced effect observed in combination with bevacizumab. Dual treatment disrupted ST3Gal1–VEGF‐A interactions and downregulated angiogenic and EMT markers.

**Conclusion:**

ST3Gal1 promotes EC progression by enhancing VEGF‐A signaling and EMT via the FAK/paxillin pathway. Its inhibition improves the efficacy of antiangiogenic therapy, supporting ST3Gal1 as a promising therapeutic target to overcome anti‐VEGF‐A resistance in advanced EC.

## 1 INTRODUCTION

Endometrial cancer (EC) is the most common gynecologic malignancy, accounting for 21% of cases, with mortality rates having doubled over the past two decades.^1^, ^2^, ^3^ Standard adjuvant chemotherapy (carboplatin plus paclitaxel) offers limited benefit for advanced‐stage or recurrent disease, and effective treatment options remain scarce.^4^, ^5^ Although bevacizumab, a vascular endothelial growth factor‐A (VEGF‐A)‐targeting antibody, has shown promise in preclinical studies, a randomized phase II trial combining bevacizumab with chemotherapy failed to significantly improve overall survival (OS) in advanced or recurrent EC.^6^ These limitations highlight the urgent need to identify molecular mechanisms underlying resistance to antiangiogenic therapy to improve treatment outcomes.^7^, ^8^

Aberrant sialylation—an abnormal form of glycosylation—increasingly emerges as a contributor to cancer progression and therapeutic resistance. Among these, ST3 beta‐galactoside alpha‐2,3‐sialyltransferase 1 (ST3Gal1) is a key α2,3‐sialyltransferase (α2,3‐ST) that modifies terminal galactose residues on glycoproteins,^9^, ^10^ and its overexpression has been linked to tumor progression and chemoresistance in gynecologic cancers.^11^, ^12^ In EC, VEGF‐A is frequently overexpressed and associated with high tumor grade, lymph node metastasis, and poor prognosis, underlining its role as a prognostic biomarker.^13^, ^14^, ^15^ However, it remains unclear whether ST3Gal1‐mediated sialylation influences VEGF‐A‐driven angiogenesis or contributes to resistance to anti‐VEGF therapy.

In this study, we hypothesized that ST3Gal1 overexpression promotes EC progression and mediates resistance to antiangiogenic therapy by modulating VEGF‐A and its downstream signaling. This study investigates the therapeutic potential of targeting the ST3Gal1/VEGF‐A axis to overcome angiogenic resistance and suppress tumor progression in EC.

## 2 MATERIALS AND METHODS

### 2.1 Cell lines and culture

Four human EC cell lines—HEC‐1A, HEC‐1B, RL95‐2, and HEC‐50B—were obtained from the American Type Culture Collection (ATCC). Cells were maintained in Minimum Essential Medium (MEM) or Dulbecco's Modified Eagle's Medium/Nutrient Mixture F‐12 (DMEM/F12) supplemented with 10% fetal bovine serum (FBS) and 1% penicillin–streptomycin at 37°C in a humidified atmosphere with 5% CO_2_. Cell line authenticity was confirmed by short tandem repeat (STR) profiling, and all experiments were performed using mycoplasma‐free cells.

### 2.2 Patient samples

Tumor tissues from 161 EC patients who received standard treatment at Taipei Veterans General Hospital (TVGH) between August 2012 and October 2020 were collected. Samples were snap‐frozen in liquid nitrogen and stored at −80°C. ST3Gal1 immunohistochemistry (IHC) was scored by a pathologist (C‐RL), with intensity categorized as weak (0–1) or strong (2, 3). Quantification was performed using ImageJ. The study was approved by the Institutional Review Board (IRB no. 2021–02‐002B).

### 2.3 Immunohistochemistry

Paraffin‐embedded tissue sections (4 μm) were deparaffinized, rehydrated, and subjected to antigen retrieval using citrate buffer (pH 6.0). After blocking with 1% bovine serum albumin, sections were incubated overnight at 4°C with primary antibodies against ST3Gal1, VEGF‐A, CD31, and epithelial–mesenchymal transition (EMT)‐related markers. Horseradish peroxidase (HRP)‐conjugated secondary antibodies were applied for 2 hours at room temperature. Signals were developed using 3,3′‐diaminobenzidine (DAB) and counterstained with hematoxylin. Images were captured using a Nikon Ci light microscope. All experiments were performed in triplicate.

### 2.4 RNA extraction and quantitative PCR

Total RNA was extracted using TRIzol reagent (Thermo Fisher Scientific, Waltham, MA, USA) and reverse‐transcribed into cDNA using the PrimeScript RT reagent Kit (Takara, Shiga, Japan) with 1 μg of RNA. Gene expression was quantified using the LightCycler 480 Real‐Time PCR System (Roche, Basel, Switzerland) and Universal Probe Library. ST3Gal1 and VEGF‐A were analyzed with β‐actin as the internal control. Relative expression was calculated using the 2^−ΔΔCT^ method. All experiments were performed in triplicate.

### 2.5 Western blot

Cells were lysed in radioimmunoprecipitation assay buffer, and protein samples (50 μg) were denatured at 95°C for 10 minutes and resolved by 6% or 10% sodium dodecyl sulphate‐polyacrylamide gel electrophoresis (SDS‐PAGE). Proteins were transferred to Immobilon membranes (Millipore, Merck KGaA, Darmstadt, Germany) via wet transfer. Membranes were blocked in 5% nonfat dry milk (0.1% Tween‐20/PBS), incubated overnight with primary antibodies, and then with HRP‐conjugated secondary antibodies. Signals were detected using enhanced chemiluminescence (Amersham, Piscataway, NJ, US) and visualized with a ultraviolet peroxide (UVP) system. Primary antibodies including ST3Gal1, VEGF‐A, E‐cadherin, N‐cadherin, vimentin, and α‐SMA (iREAL, ProteinTech, ABclonal, Hsinchu City, Taiwan) were utilized to assess EMT. Antibodies targeting focal adhesion kinase (FAK), paxillin, Cdc42, and p38—key downstream effectors of the VEGF‐A/VEGFR2 signaling axis—were used to assess the effects of VEGF‐A downstream pathways following ST3Gal1 inhibition.^16^, ^17^, ^18^, ^19^, ^20^, ^21^, ^22^ Protein expression was examined in RL95‐2 and HEC‐1B cells following either ST3Gal1 knockdown or treatment with the ST3Gal1 inhibitor SsaI. Cells were harvested 48 hours after treatment with glyceraldehyde‐3‐phosphate dehydrogenase (GAPDH) as the internal control. All experiments were performed in triplicate.

### 2.6 Colony formation assay

RL95‐2 and HEC‐1B cells (500/well) were seeded in six‐well plates and treated with SsaI (25, 50, or 100 μM) or dimethyl sulfoxide (DMSO) control. SsaI (catalog no. S9951; Sigma‐Aldrich, St Louis, MO, USA) is a specific ST3Gal1 inhibitor.^23^ Cells were cultured for 10–14 days with media changes every 3 days. Colonies (≥50 cells) were fixed with 4% paraformaldehyde, stained with 0.5% crystal violet, and quantified using ImageJ. Colony formation was normalized to the DMSO control.

### 2.7 Duolink proximity ligation assay (PLA)

Protein–protein interactions between ST3Gal1 and VEGF‐A were detected using the Duolink in situ kit (Olink Bioscience, Uppsala, Sweden) following the manufacturer's protocol. Tissue specimens were incubated with primary antibodies for 2 hours, followed by PLA probe hybridization and fluorescence detection. Images were acquired using a Nikon fluorescence microscope. Experiments were repeated in triplicate.

### 2.8 ST3Gal1 gene knockdown

Lentiviral vectors expressing short hairpin RNA (shRNA) targeting human ST3Gal1 (sequence: GCGGGAGAAGAAGCCCAATAA) and a scrambled control were provided by the RNAi Core Facility, Academia Sinica, Taiwan. Vectors were co‐transfected with packaging plasmids using Lipofectamine 2000 (ThermoFisher Scientific, Waltham, MA USA). Stable cells were selected with 1 μg/mL puromycin (Sigma‐Aldrich). Gene and protein expression were confirmed by quantitative polymerase chain reaction (qPCR) and Western blotting.

### 2.9 Transwell invasion assay

Cell invasion was assessed using 24‐well Transwell inserts (8‐μm pores; Corning, Corning, NY, USA) pre‐coated with Matrigel (0.4 mg/mL; Becton Dickinson, Franklin Lakes, NJ, USA). RL95‐2 and HEC‐1B cells (1 × 10^5^) in serum‐free medium were seeded in the upper chamber, with 20% FBS in the lower chamber as a chemoattractant. After 48 hours, non‐invading cells were removed, and invaded cells on the underside were fixed, stained with hematoxylin, and counted in 10 random fields at 40× magnification. Invasion rates were normalized to proliferation using ImageJ. Experiments were performed in triplicate.

### 2.10 Wound healing assay

RL95‐2 and HEC‐1B cells (1 × 10^5^/well) were cultured overnight in six‐well plates. Monolayers were scratched with a 200 μL pipette tip and rinsed with PBS to remove debris. Cells were then treated with SsaI and/or bevacizumab. Migration was monitored at 0 and 48 hours using a Leica DMi1 microscope with MC120 HD camera (Wetzlar, Germany). Wound closure was quantified using ImageJ: ([wound area at 0 h – wound area at 48 h]/wound area at 0 h) × 100%. All experiments were done in triplicate.

### 2.11 Lectin affinity immunoprecipitation

Cells were lysed in cold immunoprecipitation (IP) buffer (0.5% NP‐40, 5 mM EDTA, 10% glycerol, 100 mM NaCl, 50 mM Tris–HCl, pH 7.5) with protease/phosphatase inhibitors. Lysates (1 mg) were incubated with 10 μg biotinylated Maackia amurensis lectin II (MAL‐II; Vector Laboratories, Burlingame, California, US) for 3 hours at room temperature, followed by streptavidin‐agarose beads (Thermo Fisher Scientific) for 2 hours. Bound sialylated proteins were collected by centrifugation and washed with IP buffer.

### 2.12 In Vivo xenograft model

Twenty‐four female BALB/c athymic (nu+/nu+) mice (4–6 weeks) were housed under pathogen‐free conditions and acclimated for 1 week. Mice were subcutaneously injected with 6 × 10^6^ RL95‐2 or HEC‐1B cells suspended in 200 μL Matrigel (Collaborative Biomedical Products, Bedford, MA, USA). When tumors reached ~100 mm^3^, mice were randomized into four groups (*n* = 6 per group): control (0.9% saline, oral gavage), SsaI (20 mg/kg, oral daily), bevacizumab (5 mg/kg, intraperitoneally twice weekly), and combination (SsaI + bevacizumab). The dosing regimens were selected based on previously published preclinical studies demonstrating the in vivo tolerability and biological activity of oral SsaI at 20 mg/kg,^24^, ^25^ as well as the established use of bevacizumab at 5 mg/kg in xenograft models of gynecologic and other solid tumors.^26^, ^27^, ^28^ Tumor volume and body weight were recorded twice weekly. At endpoint, mice were euthanized, and tumors were harvested for volume and weight assessment. All procedures were Institutional Animal Care and Use Committee (IACUC)‐approved and conducted according to institutional ethical guidelines.

### 2.13 Statistical analysis

Statistical analyses were conducted using SPSS v26.0 and GraphPad Prism 10. Data normality was assessed using the Shapiro–Wilk test. Unpaired *t*‐tests were used for normally distributed two‐group comparisons; the Mann–Whitney *U*‐test was used for non‐parametric data. One‐way ANOVA with Bonferroni correction was applied for multi‐group comparisons. Data are presented as mean ± standard deviation from at least three independent experiments. A *P*‐value <0.05 was considered statistically significant.

## 3 RESULTS

### 3.1 ST3Gal1 facilitates tumor progression and correlates with poor prognosis in EC

**FIGURE 1:**
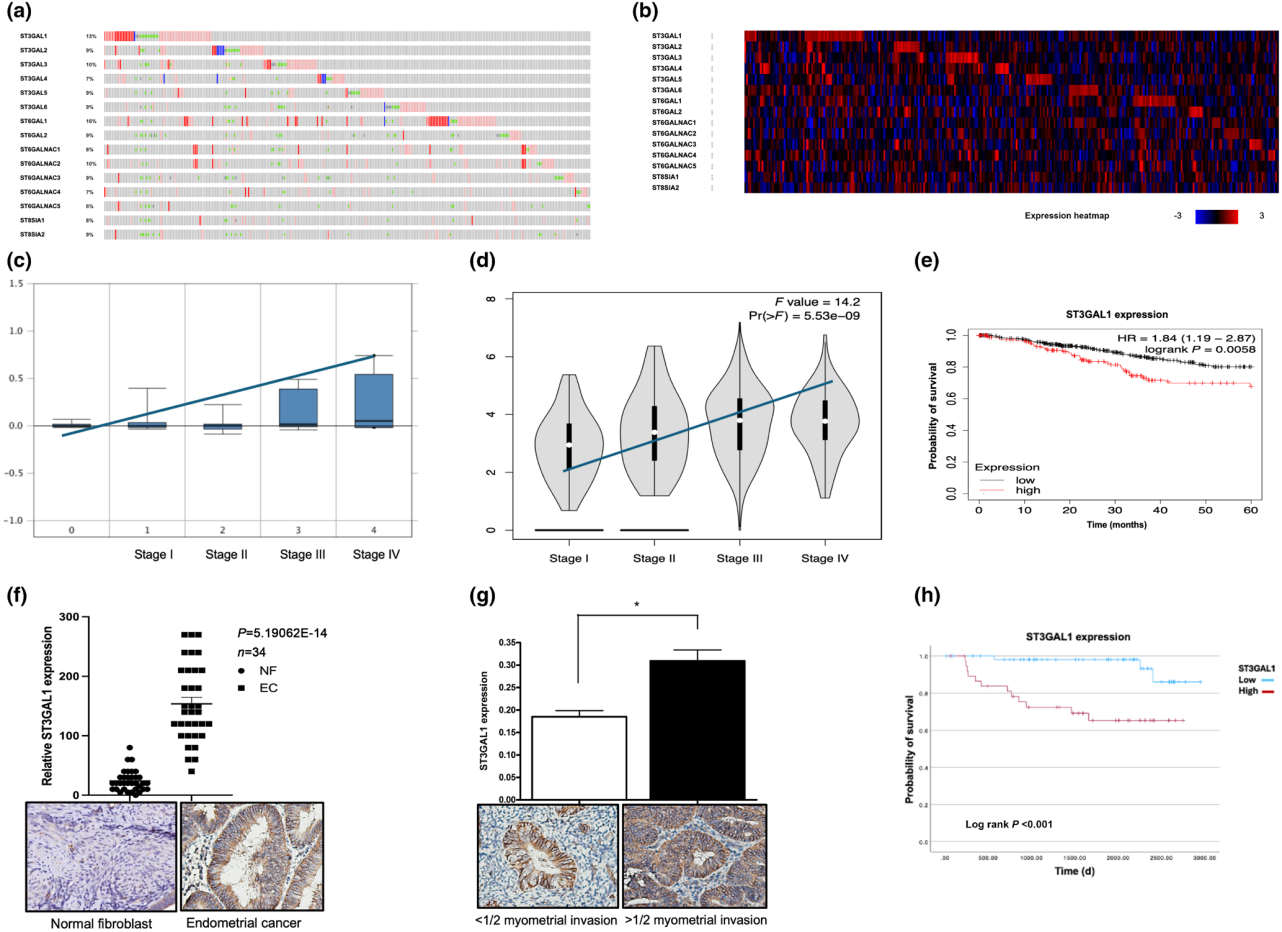
ST3Gal1 DNA amplification, mRNA overexpression, and protein upregulation drive endometrial cancer (EC) progression and poor prognosis. (a) Genomic alterations of sialyltransferase (ST) family genes in EC based on The Cancer Genome Atlas (TCGA) dataset, analyzed by cBioPortal. ST3 beta‐galactoside alpha‐2,3‐sialyltransferase 1 (ST3Gal1) and ST6Gal1 exhibit higher DNA copy number variations among all STs. (b) Heatmap showing differential mRNA expression of STs in EC samples, highlighting ST3Gal1 as the most upregulated gene. (c) Oncomine analysis demonstrating a significant correlation between increasing ST3Gal1 DNA copy number and higher FIGO (International Federation of Gynecology and Obstetrics) stage. (d) Violin plot analysis revealing a positive correlation between ST3Gal1 mRNA expression and FIGO stage in EC patients (*P* < 0.0001). (e) Kaplan–Meier survival analysis from TCGA data, indicating that high ST3Gal1 expression correlates with reduced overall survival (log‐rank *P* = 0.0058, hazard ratio [HR] 1.84, 95% confidence interval [CI] 1.19–2.87). (f) Immunohistochemical (IHC) analysis comparing ST3Gal1 protein expression in normal fibroblasts (NF) vs. EC tissues, showing significantly higher expression in EC samples (*P* < 0.0001, *n* = 68). Representative images are shown below. (g) IHC analysis of ST3Gal1 expression in EC patients with <1/2 versus >1/2 myometrial invasion, demonstrating significantly higher expression in cases with deep myometrial invasion (*P* < 0.05). (h) Kaplan–Meier survival analysis of the TVGH EC cohort (*n* = 161), confirming significantly worse survival in patients with high ST3Gal1 expression (log‐rank *P* < 0.0001).

To investigate the role of sialyltransferase (ST) in EC, we analyzed the Cancer Genome Atlas (TCGA) data using cBioPortal.^29^ Among all ST family members, ST6 beta‐galactoside alpha‐2,6‐sialyltransferase 1 (ST6Gal1) and ST3Gal1 exhibited higher frequencies of DNA copy number variation, with ST6Gal1 showing a slightly higher frequency of variations compared with ST3Gal1 (16% vs. 13%). However, ST3Gal1 demonstrated the most pronounced mRNA overexpression across EC samples (Figure 1b), supporting its functional relevance. Analysis using the Oncomine database^30^ showed that increased ST3Gal1 copy number correlated with higher FIGO (International Federation of Gynecology and Obstetrics) stage (Figure 1c), and ST3Gal1 mRNA levels were significantly elevated in advanced‐stage tumors (Figure 1d). Kaplan–Meier survival analysis of the TCGA cohort demonstrated that high ST3Gal1 expression was significantly associated with shorter overall survival (*P* = 0.0058) (Figure 1e). These findings were validated in an independent cohort of 161 EC patients from TVGH, where IHC analysis showed significantly higher ST3Gal1 expression in tumors than in normal fibroblasts (*P* < 0.0001) (Figure 1f), and in tumors with deep myometrial invasion (>1/2 wall thickness) compared with superficial invasion (<1/2 wall thickness) (*P* < 0.05) (Figure 1g). Kaplan–Meier analysis of the TVGH cohort also confirmed significantly worse 5‐year survival in patients with high ST3Gal1 expression (*P* < 0.0001) (Figure 1h). Together, these data identify ST3Gal1 as a key promoter of EC progression and a candidate prognostic biomarker.

### 3.2 ST3Gal1 regulates VEGF‐A expression, EMT, and glycosylation

**FIGURE 2:**
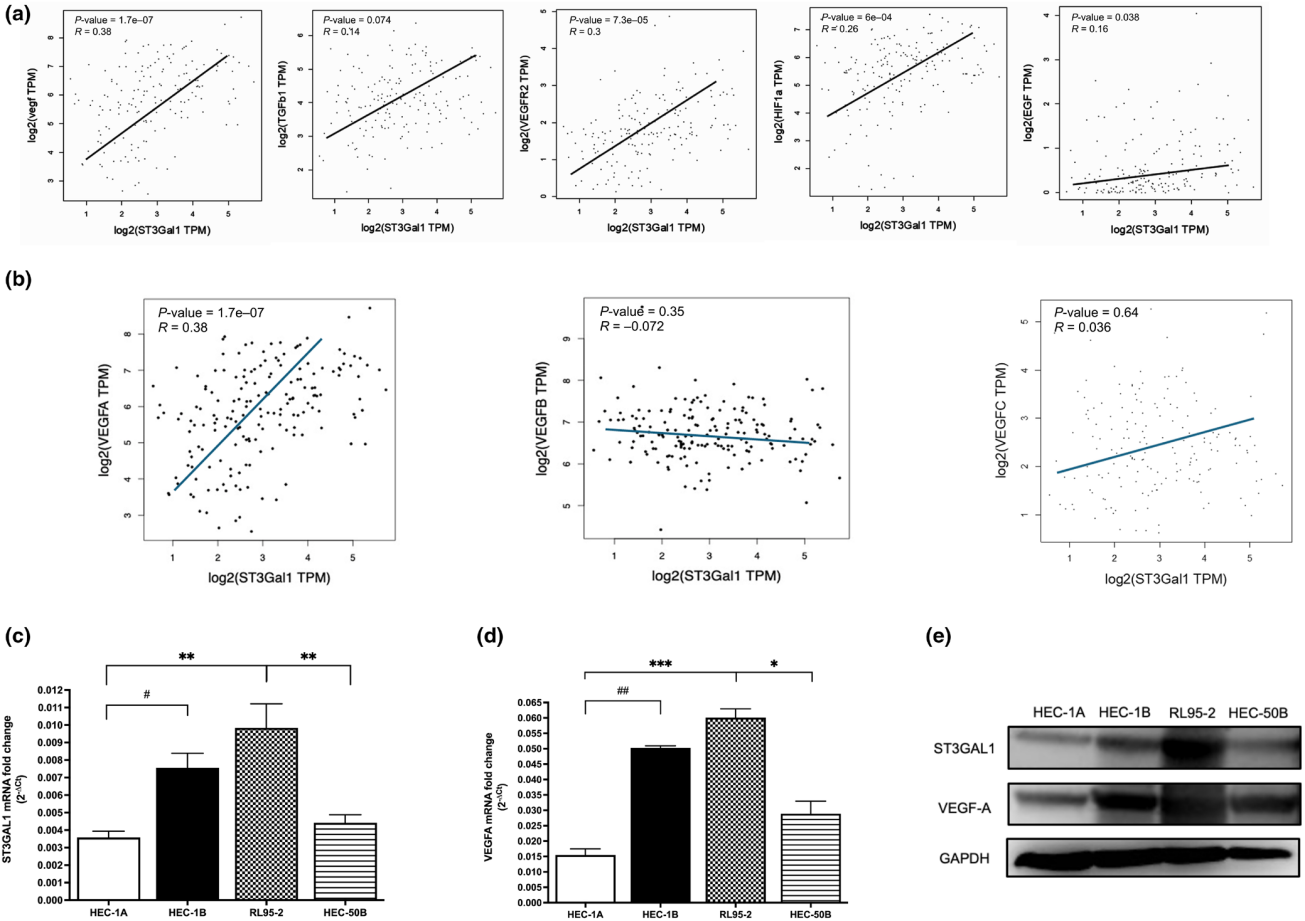
ST3 beta‐galactoside alpha‐2,3‐sialyltransferase 1 (ST3Gal1) correlates with vascular endothelial growth factor‐A (VEGF‐A) expression in endometrial cancer (EC). (a) The Cancer Genome Atlas (TCGA)‐based correlation analysis of ST3Gal1 mRNA expression with key genes involved in angiogenesis and metastasis, including VEGF, TGF‐β1, VEGFR2, HIF‐1α, and endothelial growth factor (EGF). (b) Comparative correlation of ST3Gal1 with VEGF‐A, VEGF‐B, and VEGF‐C, highlighting the strongest association with VEGF‐A. (c, d) mRNA expression levels of ST3Gal1 (c) and VEGF‐A (d) in EC cell lines (HEC‐1A, HEC‐1B, RL95‐2, HEC‐50B), with significant upregulation observed in RL95‐2 and HEC‐1B. (e) Western blot analysis of ST3Gal1 and VEGF‐A protein expression across four EC cell lines.

**FIGURE 3:**
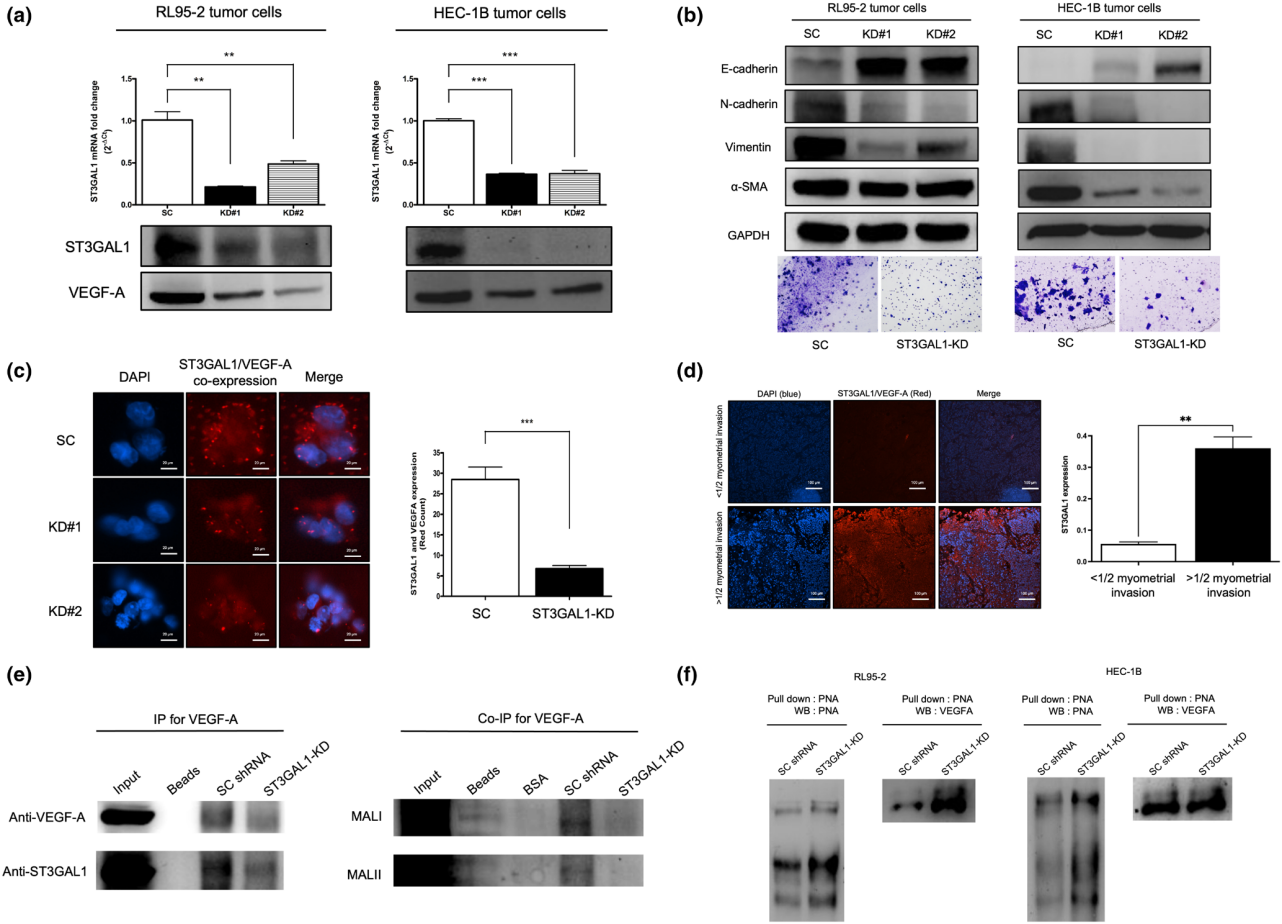
Functional impact of ST3 beta‐galactoside alpha‐2,3‐sialyltransferase 1 (ST3Gal1) knockdown (KD) on vascular endothelial growth factor‐A (VEGF‐A) Signaling and epithelial–mesenchymal transition (EMT) in endometrial cancer (EC). (a) ST3Gal1 mRNA expression in RL95‐2 and HEC‐1B cells following ST3Gal1 KD (KD#1, KD#2) compared with scrambled control (SC). Protein expression levels of ST3Gal1 and VEGF‐A are shown using Western blot. (b) Western blot analysis of EMT markers (E‐cadherin, N‐cadherin, vimentin, α‐SMA) in RL95‐2 and HEC‐1B cells following ST3Gal1 KD. Bottom panels: Representative Transwell invasion assays of control (SC) and ST3Gal1‐KD cells. (c) Duolink proximity ligation assay (PLA) demonstrating ST3Gal1 and VEGF‐A co‐expression in EC cells following ST3Gal1 KD (KD#1, KD#2) compared with scrambled control (SC). Red spots indicate protein–protein interactions, and DAPI (blue) marks nuclei. Quantification of interactions is shown on the right. (d) Duolink PLA demonstrating ST3Gal1 and VEGF‐A interactions in EC tissues, with increased interaction in tumors with >1/2 myometrial invasion compared with those with <1/2 myometrial invasion. Quantification of ST3Gal1/VEGF‐A co‐expression is shown on the right. Relative quantification is performed using ImageJ software. (e) Immunoprecipitation (IP) of VEGF‐A in RL95‐2 cells. ST3Gal1 KD reduces the VEGF‐A–ST3Gal1 interaction, as shown by decreased VEGF‐A levels in IP products compared with SC shRNA. Co‐immunoprecipitation (Co‐IP) of VEGF‐A. ST3Gal1‐KD reduces ST3Gal1 binding in VEGF‐A immunoprecipitates, indicating disruption of their interaction. (f) Peanut agglutinin (PNA) pull‐down assays in RL95‐2 and HEC‐1B cells demonstrated that ST3Gal1 KD reduced VEGF‐A sialylation, as evidenced by increased PNA binding in KD cells versus SC controls.

Given the clinical significance of ST3Gal1, we next examined its functional role in angiogenesis and metastasis. Correlation analysis showed that ST3Gal1 was most strongly associated with VEGF expression, particularly VEGF‐A, compared with other angiogenic regulators such as TGF‐β1, VEGFR2, Hypoxia inducible factor‐1 alpha (HIF‐1α), and endothelial growth factor (EGF) (Figure 2a,b). Among four EC cell lines, RL95‐2 and HEC‐1B showed the highest ST3Gal1 and VEGF‐A mRNA and protein levels (Figure 2c–e). ST3Gal1 knockdown via shRNA in these two cell lines resulted in reduced VEGF‐A protein expression (Figure 3a), and reversed EMT marker expression, increasing E‐cadherin while reducing N‐cadherin, vimentin, and α‐SMA, particularly in RL95‐2 cells. Transwell invasion assays confirmed impaired invasive capacity following ST3Gal1 knockdown (Figure 3b). Duolink PLA confirmed a reduction in ST3Gal1–VEGF‐A interactions in both knockdown cell lines (Figure 3c), and in tissue samples, tumors with deep myometrial invasion exhibited significantly higher ST3Gal1–VEGF‐A interaction than those with superficial invasion (Figure 3d). To assess whether ST3Gal1 regulates VEGF‐A via glycosylation, immunoprecipitation and Co‐immunoprecipitation assays showed reduced binding between ST3Gal1 and VEGF‐A upon knockdown (Figure 3e). Peanut agglutinin (PNA) pull‐down revealed increased PNA binding in ST3Gal1‐knockdown cells, which is consistent with reduced α2,3‐sialylation and exposure of galactose residues, indicating altered glycosylation of VEGF‐A, more prominent in RL95‐2 than in HEC‐1B cells (Figure 3f).

### 3.3 Pharmacologic inhibition of ST3Gal1 suppresses EC cell growth and EMT

**FIGURE 4:**
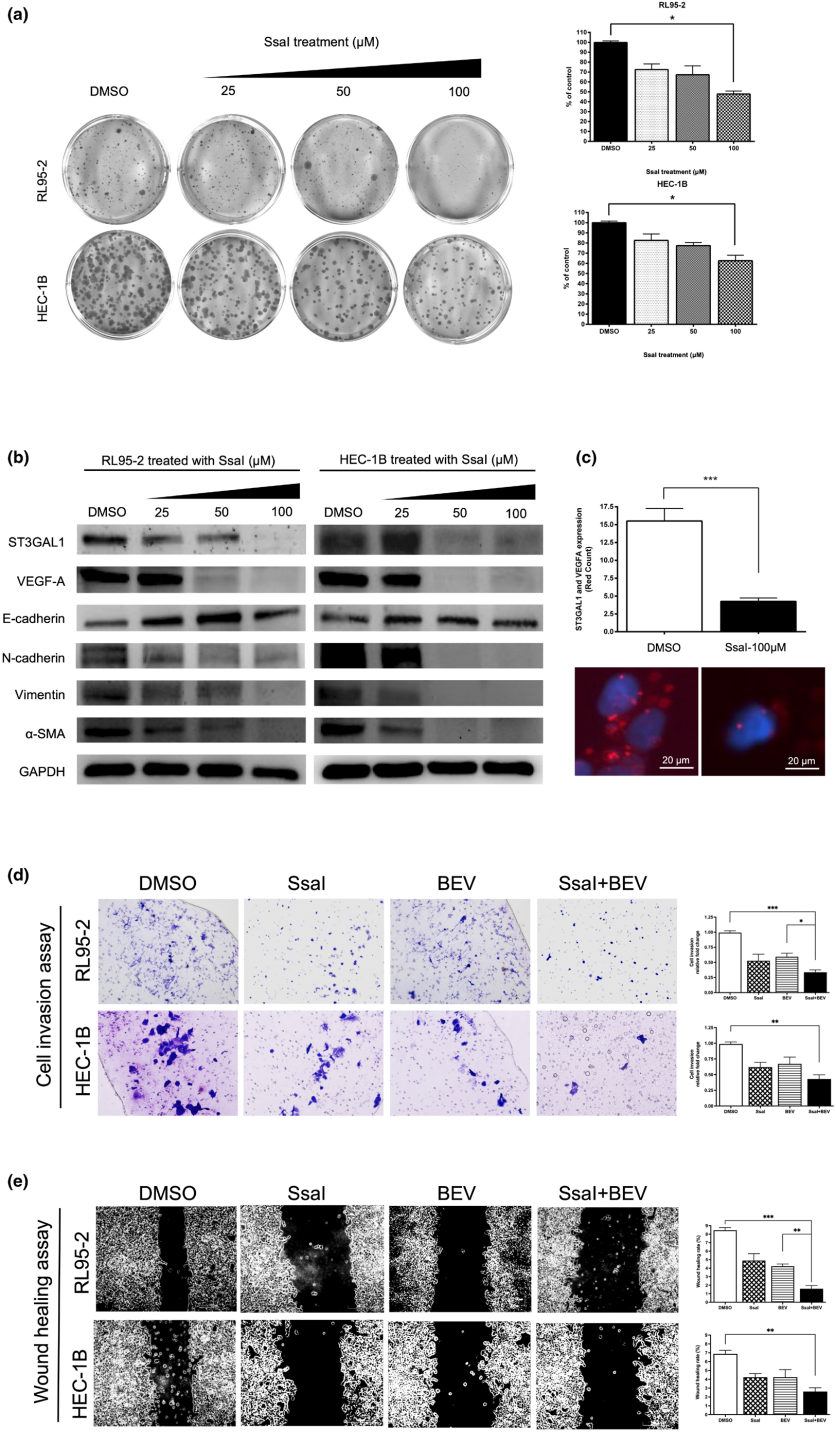
In vitro evaluation of the effect of soyasaponin I (SsaI), bevacizumab, and their combination on endometrial cancer (EC) cells. (a) Colony formation assay showing the effect of increasing concentrations of SsaI (dimethyl sulfoxide [DMSO], 25, 50, and 100 μM) on RL95‐2 and HEC‐1B cell proliferation. Quantification of colony number is presented on the right (*P* < 0.05). (b) Western blot analysis of ST3 beta‐galactoside alpha‐2,3‐sialyltransferase 1 (ST3Gal1), vascular endothelial growth factor‐A (VEGF‐A), and epithelial–mesenchymal transition (EMT) markers (E‐cadherin, N‐cadherin, vimentin, and α‐SMA) in RL95‐2 and HEC‐1B cells treated with increasing concentrations of SsaI. glyceraldehyde‐3‐phosphate dehydrogenase (GAPDH) served as a loading control. (c) Duolink proximity ligation assay demonstrating ST3Gal1 and VEGF‐A co‐expression in EC cells. Red spots indicate protein–protein interactions, and DAPI (blue) marks the nucleus. Quantification of ST3Gal1/VEGF‐A interactions is shown on the top (*P* < 0.001). (d) Transwell invasion assay of RL95‐2 and HEC‐1B cells treated with DMSO, SsaI, bevacizumab, or SsaI + bevacizumab. Cells were stained with crystal violet. Quantification of relative invasion is shown on the right. (*P* < 0.05, *P < 0.01, **P < 0.001). (e) Wound‐healing assay performed on RL95‐2 and HEC‐1B cells treated with DMSO, SsaI, bevacizumab, or SsaI + bevacizumab. Representative images of wound closure at 48 hours are shown. Quantification of wound closure rate is presented on the right (*P* < 0.05, *P < 0.01, **P < 0.001).

To evaluate the therapeutic potential of targeting ST3Gal1, RL95‐2 and HEC‐1B cells were treated with increasing concentrations of the ST3Gal1‐specific inhibitor SsaI (25–100 μM). Colony formation assays showed a dose‐dependent suppression of cell growth, with significant inhibition at 100 μM (Figure 4a). Western blot analysis confirmed dose‐dependent decreases in ST3Gal1 and VEGF‐A, alongside EMT marker reversal—upregulation of E‐cadherin and downregulation of N‐cadherin, vimentin, and α‐SMA (Figure 4b). Duolink PLA confirmed significantly reduced ST3Gal1–VEGF‐A interactions following SsaI treatment (Figure 4c), consistent with genetic knockdown results.

### 3.4 Dual targeting of ST3Gal1 and VEGF‐A synergistically inhibits cell invasion and migration

We next assessed whether combined targeting of ST3Gal1 and VEGF‐A would produce synergistic effects on EC cell invasion and migration. In Transwell assays, both SsaI and bevacizumab monotherapy significantly reduced invasion compared with the DMSO control, with the combination yielding greater inhibition in RL95‐2 cells (Figure 4d). Similarly, wound‐healing assays showed that both treatments impaired migration, with the combination further enhancing this effect, particularly in RL95‐2 cells (Figure 4e). The greater sensitivity of RL95‐2 cells may be attributed to their higher ST3Gal1 and VEGF‐A expression. These results suggest that dual inhibition of ST3Gal1 and VEGF‐A has enhanced anti‐metastatic effects in EC cells with high ST3Gal1 expression.

### 3.5 Dual inhibition suppresses tumor growth and EMT in vivo

**FIGURE 5:**
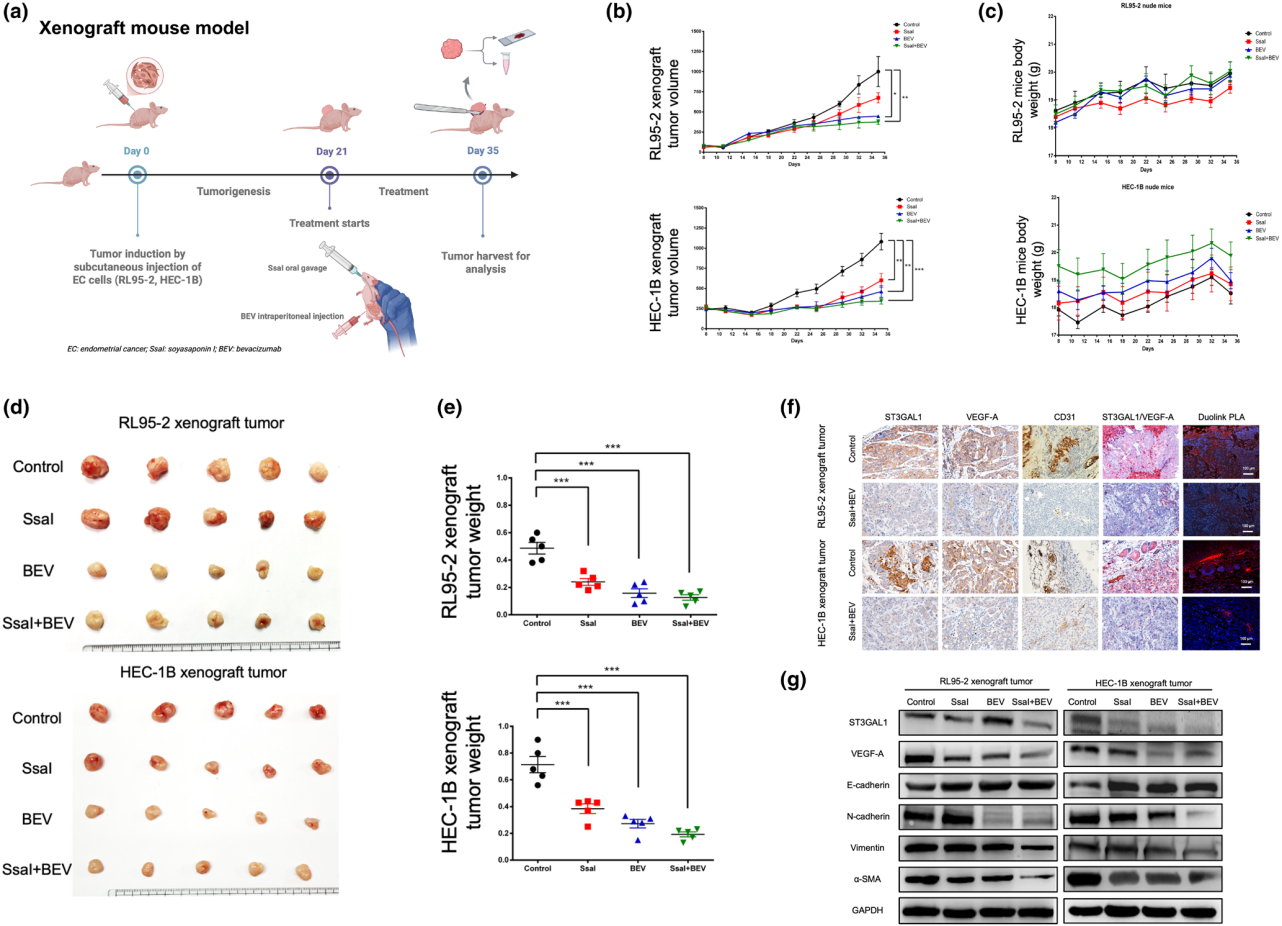
In vivo evaluation of soyasaponin I (SsaI) and bevacizumab treatment on endometrial cancer (EC) using RL95‐2 and HEC‐1B xenografts. (a) Schematic representation of the xenograft study. Created using BioRender.com. (b) Tumor volume growth curves over time for RL95‐2 and HEC‐1B xenografts in mice treated with control (black), SsaI (red), bevacizumab (BEV; blue), or the combination of SsaI and bevacizumab (SsaI + BEV; green) over the course of the experiment. (c) Body weight measurement of RL95‐2 and HEC‐1B xenograft‐bearing nude mice treated with control (black), SsaI (red), bevacizumab (BEV; blue), or the combination of SsaI and bevacizumab (green) over the course of the experiment. (d) Gross images of the harvested RL95‐2 and HEC‐1B xenograft tumors. (e) Tumor weights at the time of harvest. (f) Immunohistochemistry (IHC) analysis of ST3 beta‐galactoside alpha‐2,3‐sialyltransferase 1 (ST3Gal1), vascular endothelial growth factor‐A (VEGF‐A), and CD31 expression, along with ST3Gal1/VEGF‐A dual staining and Duolink proximity ligation assay (PLA) assays, was performed to compare the combination treatment with the control group. (f) Western blot analysis of ST3Gal1, VEGF‐A, and epithelial–mesenchymal transition (EMT) markers (E‐cadherin, N‐cadherin, vimentin, and α‐SMA) in RL95‐2 and HEC‐1B xenografts tumor cells after sacrifice.

To validate these findings in vivo, xenograft models using RL95‐2 and HEC‐1B cells were treated with vehicle, SsaI, bevacizumab, or the combination. The dual treatment significantly suppressed tumor growth and volume compared with monotherapies, with no notable impact on body weight (Figure 5a–e). IHC revealed reduced expression of ST3Gal1, VEGF‐A, CD31, and co‐localized ST3Gal1–VEGF‐A in the combination group. Duolink PLA confirmed fewer ST3Gal1–VEGF‐A interactions in tumors treated with SsaI + bevacizumab (Figure 5f). Western blot analysis showed increased E‐cadherin and decreased mesenchymal markers (Figure 5g), indicating EMT suppression and reduced metastatic potential with dual therapy.

### 3.6 ST3Gal1 inhibition disrupts VEGF‐A/FAK/paxillin signaling

**FIGURE 6:**
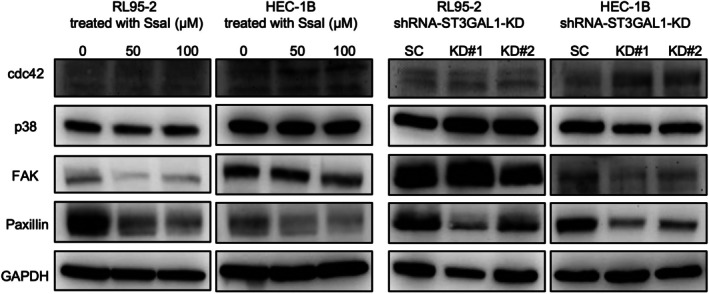
ST3 beta‐galactoside alpha‐2,3‐sialyltransferase 1 (ST3Gal1) inhibition disrupts focal adhesion kinase (FAK)/paxillin signaling. Western blot analysis of RL95‐2 and HEC‐1B cells treated with soyasaponin I (SsaI; 0, 50, or 100 μM) or ST3Gal1 knockdown (shRNA‐ST3Gal1‐KD). Protein expression of Cdc42, P38, FAK, and paxillin is examined to assess downstream signaling. FAK and paxillin expression are markedly reduced following SsaI treatment and ST3Gal1 knockdown, while Cdc42 and P38 levels remain unchanged.

To explore the mechanism by which ST3Gal1 promotes EC cell migration, we evaluated VEGF‐A downstream signaling. Western blotting showed that SsaI treatment reduced FAK and paxillin expression in a dose‐dependent manner, while Cdc42 and p38 levels were unchanged (Figure 6). Similar results were observed with ST3GAL1 knockdown, with marked suppression of paxillin and moderate reduction in FAK expression. These findings suggest that ST3Gal1 promotes EC cell motility via VEGF‐A–driven activation of the FAK/paxillin axis, and its inhibition effectively impairs this pathway.

## 4 DISCUSSION

In this study, we demonstrated that ST3Gal1, a key sialyltransferase responsible for α‐2,3 sialylation, is aberrantly expressed in EC. Although ST6Gal1 exhibited a slightly higher frequency of DNA copy number variation (16% vs. 13%), ST3Gal1 demonstrated the most pronounced mRNA overexpression among all ST family members and showed stronger associations with adverse clinical features, including higher tumor grade, advanced FIGO stage, and poorer survival outcomes. These findings support its selection as the primary focus of this study. Reverse transcription‐PCR and IHC analyses revealed significantly elevated ST3Gal1 expression in EC tissues compared with normal uterine fibroblasts, with protein levels further increased in tumors exhibiting deep myometrial invasion (>1/2 thickness) relative to superficial invasion (<1/2 thickness). These findings suggest that ST3Gal1 overexpression is associated with more aggressive, deeply invasive tumors.

In gynecologic cancers, ST3Gal1 has been shown to promote migration, invasion, and paclitaxel resistance in ovarian cancer.^31^ Our previous work further demonstrated that ST3Gal1 facilitates peritoneal dissemination via EGFR signaling, supporting a potential therapeutic role for combining α‐2,3 sialylation inhibitors with EGFR inhibitors.^10^ These findings align with our current results, indicating that ST3Gal1 enhances EC aggressiveness by promoting cell migration and invasion.

Our study also provides compelling evidence that ST3Gal1 regulates VEGF‐A expression and function, thereby promoting angiogenesis and tumor invasion. Duolink PLA revealed a close protein–protein interaction between ST3Gal1 and VEGF‐A, further supported by immunoprecipitation and co‐immunoprecipitation assays. These results reinforce the concept of glycosylation‐dependent modulation of VEGF‐A and identify ST3Gal1 as a key regulator of VEGF‐A activity. Collectively, our findings position ST3Gal1 as a promising therapeutic target to enhance anti‐VEGF therapy in EC.

Functionally, ST3Gal1 knockdown reduced EC cell migration and invasion and reversed EMT marker expression. Similarly, treatment with SsaI, a potent ST3Gal1 inhibitor,^23^ resulted in dose‐dependent suppression of migration, invasion, and EMT. Specifically, as shown in our study, RL95‐2 EC cells with a higher level of ST3Gal1 expression may be more reliant on the ST3Gal1–VEGF‐A signaling axis for maintaining invasive and migratory capabilities. Therefore, patients with high ST3Gal1‐expressing tumors may derive greater benefit from combination therapy, highlighting the potential of ST3Gal1 as a predictive biomarker for treatment response.

In vivo, combined treatment with SsaI and bevacizumab further suppressed tumor growth, reinforcing the therapeutic potential of dual inhibition. CD31 and VEGF‐A were significantly downregulated in xenografts following combination therapy. Duolink PLA and dual staining confirmed reduced ST3Gal1–VEGF‐A interaction, validating the treatment effect on angiogenesis.^32^

Cdc42 and p38 are known effectors downstream of VEGF‐A signaling, primarily involved in actin reorganization.^19^, ^22^ Paxillin, a focal adhesion adaptor protein, is also a critical downstream effector of VEGF‐A signaling and mediates FAK‐driven cell adhesion and migration.^21^ VEGF‐A has been shown to activate FAK and induce paxillin phosphorylation in endothelial cells.^33^ Given paxillin's role in FAK‐dependent cytoskeletal remodeling, its disruption may significantly impair tumor cell motility.^17^, ^20^, ^34^, ^35^ In this study, we included Cdc42, p38, paxillin, and FAK in the Western blot panel to assess which pathways are influenced by ST3Gal1 inhibition. Our findings demonstrated that ST3Gal1 inhibition selectively impairs VEGF‐mediated activation of FAK and paxillin but does not affect Cdc42 or p38 signaling, which suggests that ST3Gal1 regulates angiogenic signaling primarily through focal adhesion‐related pathways rather than cytoskeletal remodeling. Taken together, this study confirmed that both ST3GAL1 knockdown and SsaI treatment reduced paxillin expression, disrupted FAK–paxillin signaling, suppressed EMT, and impaired EC cell migration.

One limitation of the current study is the use of a standard therapeutic dose of bevacizumab (5 mg/kg) in the xenograft mouse model, which may have masked the additional benefit of ST3Gal1 inhibition when combined with SsaI. Future studies employing reduced‐dose bevacizumab regimens could help to better delineate the specific contribution of SsaI and clarify the additive or synergistic effects of dual targeting.

Recent evidence also highlights a novel immunomodulatory role of ST3Gal1 through the regulation of glyco‐immune checkpoints. In prostate cancer, ST3Gal1 was shown to promote immune evasion by synthesizing α2,3‐sialylated ligands for Siglec‐7 and Siglec‐9 receptors on immunosuppressive macrophages, thereby disrupting anti‐tumor immunity.^36^ These findings from sex‐hormone‐dependent cancer underscore the broader relevance of ST3Gal1 in both tumor‐intrinsic signaling and immune evasion.

In conclusion, ST3Gal1 promotes EC progression by modulating VEGF‐A signaling and paxillin‐mediated EMT. Its inhibition suppresses tumor growth, invasion, and angiogenesis, while enhancing the efficacy of bevacizumab. Therefore, targeting ST3Gal1 represents a promising strategy to overcome antiangiogenic resistance and warrants further clinical investigation.

## AUTHOR CONTRIBUTIONS

C‐HL: conceptualization; investigation; methodology; data curation; formal analysis; validation; writing—original draft. S‐TY: investigation; validation. W‐TC: Investigation; validation. C‐HL: methodology; data curation; visualization. Y‐CL: methodology. C‐RL: investigation; visualization. S‐LH: supervision. L‐WW: visualization. P‐HW: conceptualization; funding acquisition; methodology; formal analysis; supervision; resources; project administration; writing—review and editing.

## CONFLICT OF INTEREST STATEMENT

The authors have no conflicts of interest.

## Data Availability

Data supporting the findings of this study are available from the corresponding author upon request.
